# Morphological features predicting in-stent stenosis after pipeline implantation for unruptured intracranial aneurysm

**DOI:** 10.3389/fneur.2023.1121134

**Published:** 2023-05-12

**Authors:** Hengwei Jin, Jian Lv, Conghui Li, Jiwei Wang, Yuhua Jiang, Xiangyu Meng, Youxiang Li

**Affiliations:** ^1^Department of Neurosurgery, Beijing Tiantan Hospital and Beijing Neurosurgical Institute, Capital Medical University, Beijing, China; ^2^Department of Neurointerventional Engineering and Technology, Beijing Engineering Research Center, Beijing, China; ^3^Department of Neurosurgery, Beijing Neurosurgical Institute and Beijing Tiantan Hospital, Capital Medical University, Beijing, China; ^4^Department of Neurosurgery, The First Hospital, Hebei Medical University, Shijiazhuang, China

**Keywords:** in-stent stenosis (ISS), pipeline, radiomics, unruptured intracranial aneurysm (UIA), morphology

## Abstract

**Purpose:**

Elongation denotes the regularity of an aneurysm and parent artery. This retrospective research study was conducted to identify the morphological factors that could predict postoperative in-stent stenosis (ISS) after Pipeline Embolization Device (PED) implantation for unruptured intracranial aneurysms (UIAs).

**Methods:**

Patients with UIA and treated with PED at our institute between 2015 and 2020 were selected. Preoperative morphological features including both manually measured shape features and radiomics shape features were extracted and compared between patients with and without ISS. Logistic regression analysis was performed for factors associated with postoperative ISS.

**Results:**

A total of 52 patients (18 men and 34 women) were involved in this study. The mean angiographic follow-up time was 11.87 ± 8.26 months. Of the patients, 20 of them (38.46%) were identified with ISS. Multivariate logistic analysis showed that elongation (odds ratio = 0.008; 95% confidence interval, 0.001–0.255; *p* = 0.006) was an independent risk factor for ISS. The area under the curve (AUC) of the receiver operating characteristic curve(ROC) was 0.734 and the optimal cut-off value of elongation for ISS classification was 0.595. The sensitivity and specificity of prediction were 0.6 and 0.781, respectively. The ISS degree of elongation of less than 0.595 was larger than the ISS degree of elongation of more than 0.595.

**Conclusion:**

Elongation is a potential risk factor associated with ISS after PED implantation for UIAs. The more regular an aneurysm and parent artery, the less likelihood of an ISS occurrence.

## Introduction

The flow diverter service (FDS) has revolutionized the concept of interventional treatment of unruptured intracranial aneurysms (UIAs) by placing more emphasis on parent artery reconstruction (1). With its great ability to control blood flow direction, FDS can promote spontaneous thrombus formation within the aneurysm and facilitate the growth of vessel endangium (2). Pipeline Embolization Device (PED) was the first FDS and owing to its safety and efficacy, it is widely used for the treatment of wide-necked, large, and giant aneurysms (3, 4). However, ischemic and hemorrhagic complications of approximately 4.7% and 2.4%, respectively, deserve further attention (5).

In recent studies, in-stent stenosis (ISS) was reported in UIA patients after PED treatment, with the reported occurrence ranging between 9.8% and 61.5% (6–9). The mechanisms include inadequate antiplatelet therapy (10), aberrant endothelial or smooth muscles cells hyperplasia caused by inflammation response (2), parent artery tortuosity (11, 12), and parent artery sharp bend (13). Although parent artery morphology is an important reason for ISS, no study has yet used radiomics features to explore the potential mechanisms for ISS after PED implantation. Radiomics was first proposed by Dutch scholars in 2012 and was considered an approach to extract spatial features from patients’ particular imaging data (CT, MRI, and PET) to assist physicians with clinical decisions (14). It is an increasingly important area within the field of cancer and vascular disease (15, 16). In recent years, radiomics has been used extensively to explore the stability of UIAs (17). However, there is a lack of studies using morphological and radiomics features to predict ISS after treatment by PED for UIAs. Hence, we conducted a retrospective study to explore whether the morphological features would predict ISS after PED implantation for UIAs.

## Materials and methods

### Patients

This retrospective study reviewed patients with UIAs who underwent PED treatment at our institution from January 2015 to December 2020. We included patients who underwent at least one digital subtraction angiography (DSA) at 6 months and 12 months after PED treatment. Patients with anterior or posterior aneurysms smaller than 25 mm in diameter and treated using PED were included. The exclusion criteria were as follows: *in situ* stenosis at the distal or proximal part of the aneurysm within the distance of PED coverage; patients with macroscopic inadequate expansion or poor apposition to the vessel wall in any part of the stent; patients with diabetes (random blood glucose>11.1 mmol/L or fasting blood glucose>7.0 mmol/L) and/or poorly controlled hypertension (>140/90 mmHg); patients without sufficient follow-up imaging or clinical data; and patients who refused to participate and sign the consent form.

### Endovascular treatment procedure and antiplatelet regimen

All patients were premedicated for five or more continuous days with oral anti-platelet therapy (aspirin 100 mg/day; clopidogrel 75 mg/day), orally endovascular surgery, and underwent platelet inhibition ratio and *CYP2C19* genotype testing. For patients with unsatisfactory platelet inhibition rate or clopidogrel resistance genotype (intermediate metabolic or poor metabolic type), ticagrelor was chosen as the candidate therapy. The platelet inhibition ratio wass guaranteed before the operation for all patients. All endovascular procedures were performed under general anesthesia and according to the standard procedure of our institution. Dual antiplatelet therapy was prescribed for 6 months, and aspirin alone was prescribed for 12 months after the procedure.

### ISS definition

ISS is defined as any loss of parent artery diameter after treatment, which is shown in the DSA as a discernible gap between the vessel lumen filled with contrast agent and the metallic struts. For discernible gaps, we measured the diameter of the contrast-filled vessel and the diameter of the stent at its corresponding position. The rate of stenosis was then calculated as 1-(vessel diameter/stent diameter) × 100% (18). For the same lesion, we selected the maximum stenosis percentage for inclusion. ISS was then graded as mild (25%–50%), moderate (50%–75%), or severe (>75%). Hyperplasia was defined when there was less than 25% vessel lumen narrowing (6). The assessment and measurement of ISS were performed by a neuroradiologist with 3 years experience through DSA follow-up images and were reviewed by another senior neuroradiologist.

### Radiomics features extraction

**Figure 1:**
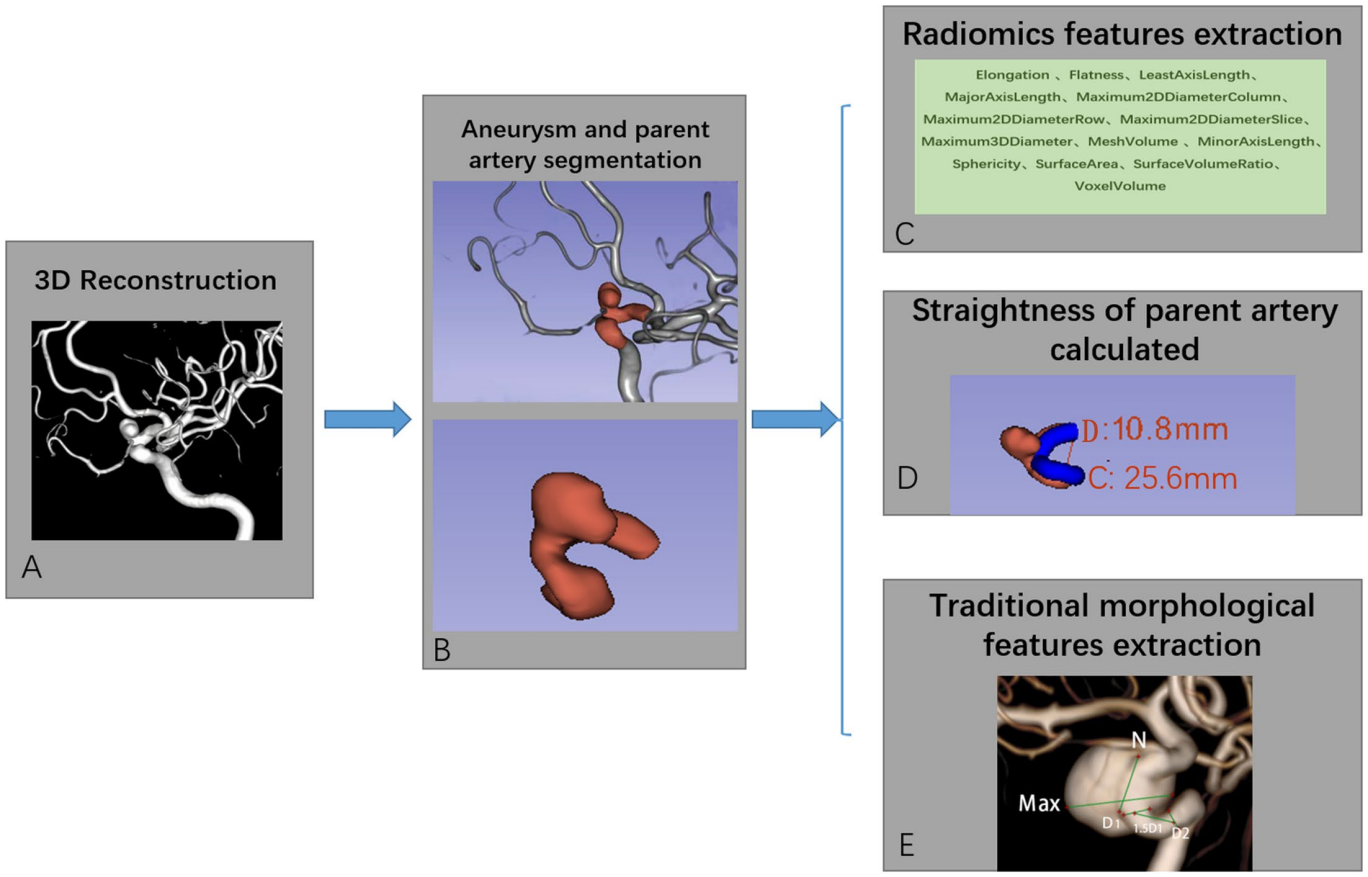
A flow chart for aneurysm and parent artery morphological features extraction. Original digital subtraction angiography data and DICOM files were used to generate a 3D reconstruction image using a 3D Slicer **(A)**. The boundaries of the region of interest (ROI) were manually segmented based on the position of the PED in the parent artery **(B)**. PyRadiomics were used to extract the radiomics features of ROI **(C)**. Tortuosity and morphological features of the aneurysm were measured **(D,E)** using the 3D Slicer.

The original Digital Imaging and Communications in Medicine (DICOM) files were obtained and UIAs with their parent artery were reconstructed using a 3D Slicer (Figure 1A). The mask of the region of interest (ROI) was manually segmented for both UIAs and their parent arteries and preserved in NIfTI files for radiomics features extraction (Figure 1B). The PyRadiomics toolkit implemented in Python was used to extract radiomics features, and this radiology quantification platform was used to set a reference standardization for feature definition and image processing (19). In consideration of other intensity-derived features that are not relevant to angiographic morphology analysis (20), only morphological features were preserved for further analysis. In this study, 14 radiomics morphological features were extracted: Elongation, Flatness, MeshVolume, Sphericity, Maximum3DDiameter, Maximum2DDiameterSlice, Maximum2DdiameterColumn, Maximum2DDiameterRow, MajorAxisLength, MinorAxisLength, LeastAxisLength, SurfaceArea, VoxelVolume, and SurfaceVolumeRatio (Figure 1C). Detailed information on these features is available in the documentation for PyRadiomics.^1^ The values of each feature for separate groups are listed in Table 1, and the flow chart of the detailed processing of images is shown in Figure 1.

**Table 1:** Radiomics morphological features.

Parameter	All	ISS	Non-ISS	*P* value
Elongation (mean ± SD)	0.63 ± 0.21	0.54 ± 0.21	0.69 ± 0.16	0.003*
Flatness (mean ± SD)	0.44 ± 0.19	0.38 ± 0.18	0.52 ± 0.18	0.004*
Least AxisLength (mean ± SD, mm)	10.64 ± 4	9.93 ± 3.93	11.43 ± 4.00	0.073
MajorAxisLength (mean ± SD, mm)	27.52 ± 14.58	29.42 ± 12.46	25.4 ± 16.63	0.095
Maximum2DDiameterColumn (mean ± SD, mm)	25.68 ± 11.1	25.4 ± 9.32	26 ± 12.98	0.927
Maximum2DDiameterRow (mean ± SD, mm)	32.51 ± 24.27	40.52 ± 30.5	23.58 ± 8.52	0.428
Maximum2DDiameterSlice (mean ± SD, mm)	31.04 ± 20.07	34.19 ± 24.12	27.54 ± 13.92	0.838
Maximum3DDiameter (mean ± SD, mm)	48.79 ± 34.37	56.48 ± 39.5	40.21 ± 25.64	0.441
MeshVolume (mean, mm^3^)	1758.79 ± 1757.3	1614.07 ± 1789.32	1920.22 ± 1741.56	0.017*
MinorAxisLength (mean, mm)	15.64 ± 6.62	15.63 ± 7.82	15.67 ± 5.13	0.622
Sphericity (mean ± SD)	0.55 ± 0.09	0.55 ± 0.87	0.56 ± 0.99	0.253
SurfaceArea (mean, mm^2^)	1178.73 ± 726.4	1089.87 ± 660.45	1277.85 ± 794.88	0.040*
SurfaceVolumeRatio (mean ± SD)	0.92 ± 0.38	0.95 ± 0.32	0.9 ± 0.44	0.019*
VoxelVolume (mean ± SD, mm^3^)	1762.55 ± 1757.04	1618.7 ± 1790.27	1923 ± 1740.08	0.017*

ISS: in-stent stenosis; SD: standard deviation. *mean statistically significant.

### Morphological parameters of an aneurysm and parent artery

Three-dimensional (3D) reconstruction angiography of the cerebral artery was built and then the segment of the parent artery covered by PED was preserved for measurement. The curve maker module of the open-source software 3D Slicer (19) (version 4.10.2; https://www.slicer.org/) was used to generate a consistent curve on the 3D mask. The length of this curve was manually measured as the centerline, which represented the length of the parent artery covered by PED. The distance between the two endpoints of this curve was measured as the distance, which represents the straight-line distance between the head and end of the parent artery covered by the PED stent. We defined the tortuosity of this parent artery in-stent segment as tortuosity = centerline (C)/Distance (D) (Figure 1D). This value implied that as the number decreased, the degree of vessel tortuosity increased. Another morphological feature described the parent artery as the mean diameter of the vessel lumen. This value was measured by averaging the diameter of the cross-section of a vessel (D1) just proximal to the neck of the aneurysm and the diameter of the cross-section (D2) at 1.5-times D1 from the neck of the aneurysm (Figure 1E). The diameter of the aneurysm neck (N) and the maximum diameter of the aneurysm (Max) were measured from the 3D reconstruction image. The schematic diagram for measuring these parameters is shown in Figure 1.

### Statistical analysis

Continuous variables were presented as means and standard deviation, and categorical variables were presented as frequencies. Univariate analysis was performed by unpaired *t*-tests and chi-squared tests for continuous variables and categorical variables, respectively. Factors with *p* < 0.05 in the univariate analysis were entered into binary multivariate logistic regression analysis, and *p* < 0.05 was considered statistically significant. We then used the Youden index to generate the optimal cut-off values of statistically significant continuous variables in the multivariate analysis. Boxplots were plotted to present the distribution of the ISS degree of the ISS group dichotomized by the optimal cut-off values. All these statistical analyses and figures were performed by SPSS and GraphPad Prism.

## Results

### Demographics and aneurysm characteristics

A total of 52 patients (18 [34.62%] men and 34 [65.38%] women, mean age: 51.02 ± 12.6 years) were included in our study. The mean DSA follow-up time was 11.87 ± 8.26 months. A total of 52 aneurysms were treated with 54 PEDs. Of the total, 44 (75%) IAs were located in the anterior circulation and eight (25%) were located in the posterior circulation. In terms of morphology, there were 41 (79.7%) saccular, three (5.1%) fusiform, and eight (15.3%) dissecting aneurysms. In addition, 35 (67.31%) aneurysms were small or medium-sized (<15 mm), and 17 (32.69%) were large aneurysms (15–25 mm). The diameter of the UIA neck ranged from 2.41 to 28.5 mm (8.74 ± 5.33 mm).

**Figure 2:**
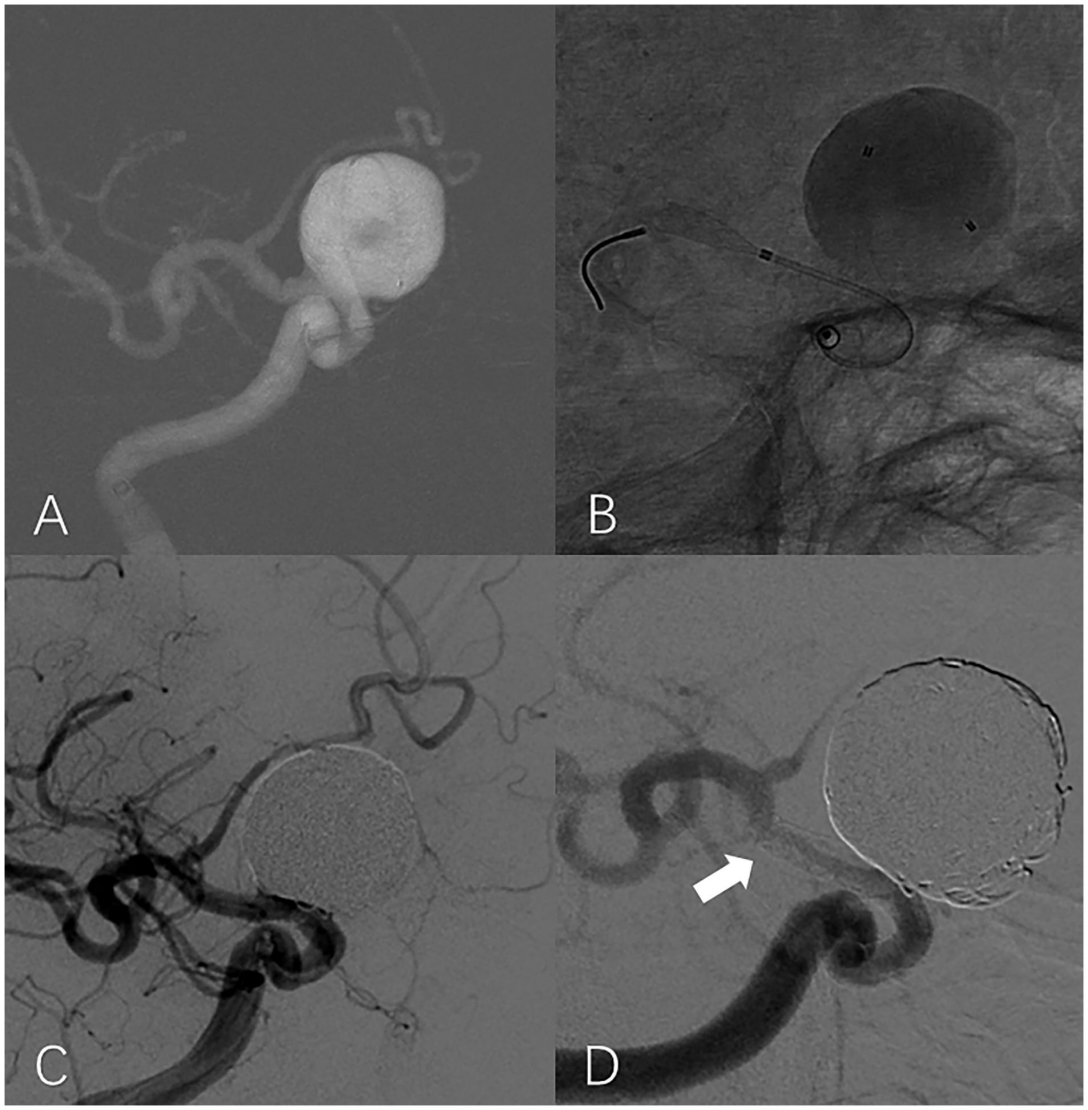
In-stent stenosis in a man in his 40s with a large aneurysm treated with PED. Roadmap shows a large unruptured aneurysm located at the supraclinoid segment of the right internal carotid artery **(A)**. Intraoperative unsubtracted angiographic image shows that the end of the PED is located at the proximal region of the ICA bifurcation **(B)**. Working projection angiography immediately after the procedure shows that the aneurysm is completely embolized and the lumen of the parent artery is well filled by contrast **(C)**. Working projection angiography at the 9-month follow-up shows the occurrence of severe ISS at the distal part of the PED (white arrow). The aneurysm is occluded without reoccurrence **(D)**.

A total of 20 (38.46%) patients were identified as having ISS; an illustrative case is presented in Figure 2. The mean angiographic follow-up period was 9.6 ± 7.3 months for the ISS group and 13.28 ± 8.62 for the non-ISS group. The ISS degree was mild in 19 (95%) patients, and serious in one patient (5%). The tortuosity of the ISS group was significantly lower than that of the non-ISS group (1.78 ± 0.62 vs. 2.29 ± 0.89, *p* = 0.03). Clinical data and IAs shape features of the two groups are presented in Table 2. Only one patient with ISS had related symptoms during the follow-up. This patient had 39% ISS at 6 months after surgery without any symptoms but suffered a sudden contralateral limb weakness at 15 months after treatment, and the ISS degree of this patient at the 21-month DSA follow-up was 30%. We observed that the ISS of this patient did not show prominent recovery, but all symptoms showed complete resolution after medical treatment. All patients with ISS underwent regular clinical and angiographic follow-ups. The platelet inhibition rate was reexamined and dual-antiplatelet therapy was extended if necessary. Either CTA or DSA was performed every 6–12 months as required.

**Table 2:** Patients’ clinical data and UIA shape parameters.

Parameters	All (*n*, %)	ISS (*n*, %)	Non-ISS (*n*, %)	*P* value
Age (mean ± SD, years)	51.02 + 12.6	51.2 ± 12.8	52.9 ± 12.7	0.936
**Sex**	0.963
Male	18	7	11	
Female	34	13	21	
**Treatment modality**	0.053
PED	25	13	12	
PED and coils	27	7	20	
**Location of IAs**	0.738
Anterior circulation	44	16	28	
Posterior circulation	8	4	4	
**IAs in the anterior circulation**	0.33
ICA	40(90.91%)	13(29.55%)	27(61.36%)	
MCA and ACoA	4(9.09%)	3(6.82%)	1(2.27%)	
**IAs in the posterior circulation**	0.429
V4 of vertebral artery	6(75%)	4(50%)	2(25%)	
Basilar artery	2(25%)	0	2(25%)	
Morphology parameters of IAs
Aneurysm neck (mean ± SD, mm)	8.74 ± 5.33	7.33 ± 4.67	9.63 ± 5.58	0.132
Aneurysm maximum diameter (mean ± SD, mm)	11.3 ± 6.16	9.22 ± 5.61	12.6 ± 6,021	0.053
Morphological parameters of the parent artery
Parent artery diameter (mean ± SD, mm)	3.78 ± 0.89	3.49 ± 0.83	3.96 ± 0.88	0.06
Tortuosity(mean ± SD)	2.09 ± 0.83	1.78 ± 0.62	2.29 ± 0.89	0.03*

UIA: unruptured intracranial aneurysm; SD: standard deviation; ISS: in-stent stenosis; PED: pipeline embolization device; MAC: middle cerebral artery; ACoA: anterior communicating artery. *mean statistically significant.

### Features predicted ISS after PED implantation and the optimal cut-off value

A total of 14 radiomics morphological features were extracted (Table 1). These features described the characteristics of the UIA and parent artery. Elongation, Flatness, SurfaceArea, MeshVolume, and VoxelVolume of the ISS group were significantly smaller than that of the non-ISS group, and the SurfaceAreaRatio of the ISS group was significantly larger than that of the non-ISS group.

**Figure 3:**
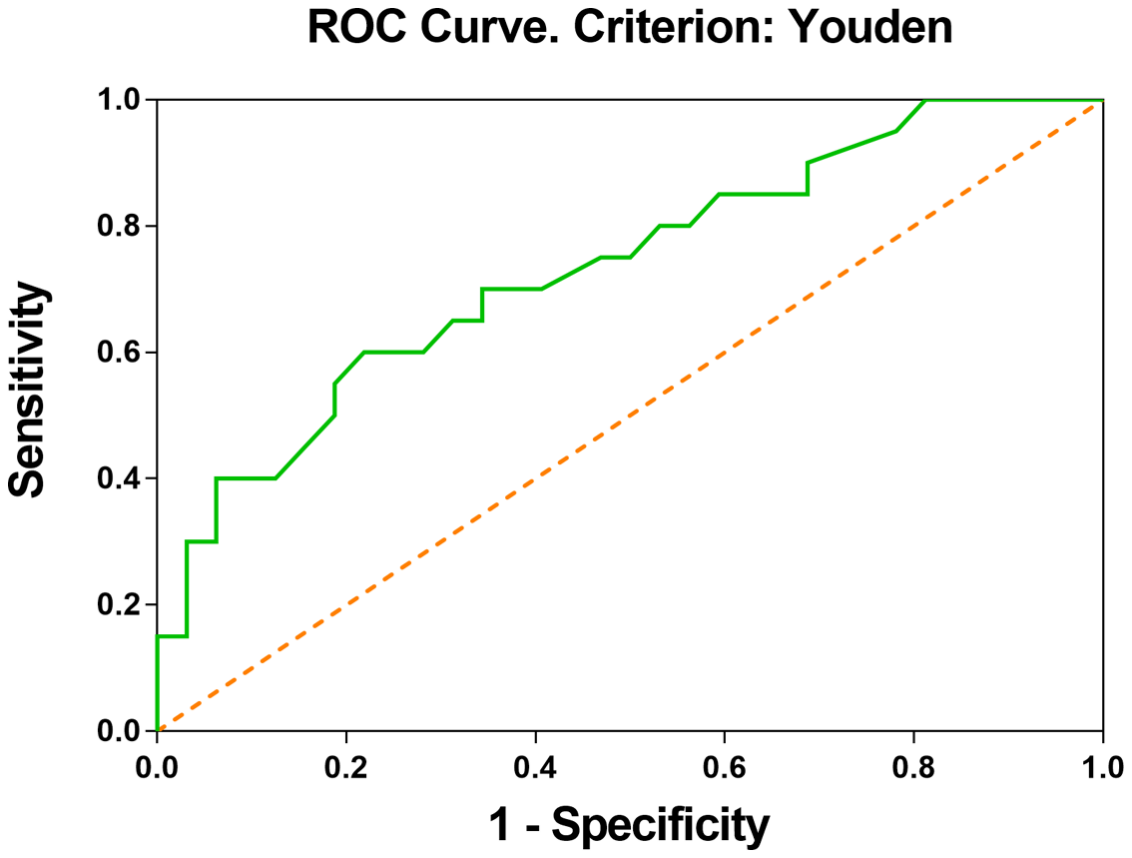
ROC curve shows that elongation is good at classifying the ISS status after PED implantation. The optimal cut-off value was calculated by the Youden index.

**Figure 4:**
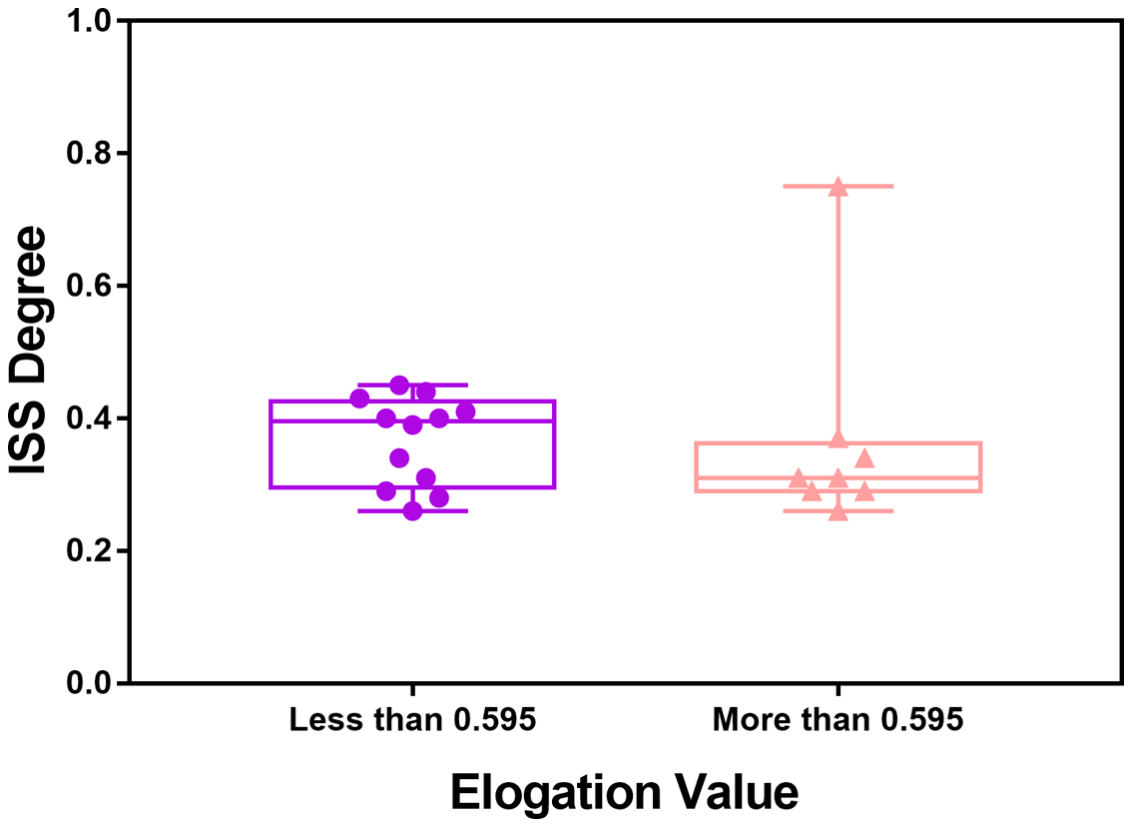
Boxplot of the value of ISS degree in the ISS group dichotomized by the optimal cut-off value of elongation.

In univariate analysis, a total of seven factors were significant, namely Tortuosity, Elongation, Flatness, MeshVolume, SurfaceArea, SurfaceVolumeRatio, and VoxelVolume. According to the definition of these features, MeshVolume and VoxelVolume both stand for the volume of ROI. We excluded the feature VoxelVolume because it is a less precise approximation of the volume than MeshVolume, and there is collinearity between the two features. The result of multivariate analysis showed that the radiomics feature Elongation (odds ratio = 0.008; 95%CI: 0.001–0.255; *p* = 0.006) was the independent factor associated with ISS (Table 3). The Elongation of the ISS group was significantly lower than the Elongation of the non-ISS group. The receiver operating characteristic curve (ROC) is presented in Figure 3. The area under the curve (AUC) of ROC was 0.734 and the optimal cut-off value was 0.595. The sensitivity and specificity of prediction were 0.6 and 0.781, respectively. The boxplot of two ISS groups dichotomized according to the optimal cut-off value of Elongation showed that the ISS degree of the group with Elongation of less than 0.595 (median, 39.5%; IQR: 29.5–42.5%) was larger than the ISS degree of the group with Elongation of more than 0.595 (median, 31%; IQR: 29–36.25%) (Figure 4).

**Table 3:** Binary logistic regression analysis.

Variable	*P* value	OR	95% CI
Elongation	0.006*	0.008	0.001–0.255
Tortuosity	0.145	0.495	0.192–12.275
MeshVolume	0.191	0.999	0.999–1
SurfaceArea	0.401	1.001	0.998–1
Flatness	0.547	8.274	0.009–7963.511
SurfaceVolumeRatio	0.926	0.789	0.005–118.268

*mean statistically significant.

## Discussion

Postoperative ISS of PED requires more attention for its high incidence rate (8). It is important to explore factors associated with ISS after implantation of PEDs for UIAs so that we can adjust the treatment plan accordingly to avoid the phenomenon and related ischemic events. Scholars have claimed that stent material, vessel hemodynamics, and parent artery morphology play an important role in this process (12, 13, 21, 22). The underlying mechanism of PED for UIAs is by promoting vessel wall reconstruction, but excessive hyperplasia can result in ISS (2). Dowlati et al. (10) claimed that ISS was related to stent thrombus and pathology of endo-vessel inflammation and demonstrated that cilostazol and clopidogrel dual anti-platelet therapy has potential efficacy in treating ISS after treatment of intracranial flow diversion, which was in accordance with results published by Cohen et al. (8). In the present study, we explored the risk factors associated with ISS using demographics and morphological features. The cohort consisted of 52 UIA patients treated by PED and the result showed that one of the radiomics features, namely, Elongation,was the independent factor for predicting ISS. In this study, all patients in this cohort were excluded from the common causes of stenosis, such as antiplatelet insufficiency, platelet resistance, and inadequate expansion or poor apposition to the vessel wall in any part of the stent. The causes of stenosis have been analyzed in terms of the morphology of UIA and its parent artery. This uncovered the high-dimensional features related to ISS and could help us in patients’ classification.

The interval of ISS occurrence seems regular. Cohen et al. (8) showed that 13 of 34 (38.2%) UIA patients treated with PED or Silk Flow Diverter stents developed signs of ISS at the first 2 months of follow-up, and all of these ISS were partially reversible with conservative measures at 20 months follow-up. The researchers thought that ISS was related to the stent creeping phenomenon that was associated with the rotational component of stents and a lower-radical stent force. Additionally, Wang et al. (9) found that ISS after PED treatment occurred at a rate of 5% (6/118 patients) in the 6–12 month follow-up, and they considered malposition of the stent, inconsistent compliance between artery and stent, and diffuse intimal hyperplasia inside the stent as the reasons for ISS. They hypothesized that stent mechanical vascular injury stimulated the vessel wall and blood cells, thus trigging cell reaction and causing platelet activation or inflammation. In addition, Ravindran et al. (18) reported that 12 of 155 (7.1%) patients were detected with ISS 6 months after the procedure, and similar to Wang et al. proposed that ISS was related to mechanical or material stimulation of the stent and subsequent inflammatory cell responses that led to cell migration.

Most ISS cases are asymptomatic self-limiting processes (7, 22). In the study by Cohen et al. (8), 12 of 13 patients with ISS were asymptomatic, while only one patient who was treated by Silk stent presented with severe distal end tapering as well as multifocal ISS in the 2 month follow-up. This patient also suffered transient ischemic attacks and achieved quick recovery under diverter angioplasty and medication during the follow-up. Ravindran et al.’s study (18) found that patients treated with PED who developed ISS had no related symptoms. However, Wang et al. (9) found that three of six ISS cases were symptomatic within 6 months of follow-up; two patients presented dizziness and one had blurring of vision. Therefore, ISS occurrence peaks at the 6-month follow-up, then most ISS will get released, although some patients may develop more serious ISS in the future. In this cohort, one patient with ISS was symptomatic. This patient experienced sudden contralateral limb weakness 15 months after treatment. An angiogram confirmed the existence of ISS. We noted that the ISS of this patient did not show prominent recovery based on the follow-up DSA at 21 months. However, all symptoms showed complete resolution after medical treatment. We suggested that ISS-induced blood flow reduction of the target area may have contributed to these symptoms.

In this cohort, Elongation was the independent factor associated with postoperative ISS in multivariate analysis. The elongation was defined as the square root of the quotient of λ-minor to λ-major, where λ-major and λ-minor were the lengths of the largest and second-largest principal component axes of the ROI, respectively (23). Elongation ranged from 1 (non-elongated) to 0 (maximally elongated, like a one-dimensional line), with convergence to 1 indicating that the ROI was nearly a circle and convergence to 0 indicating that the ROI was nearly a straight line. Elongation reflected the degree of regularity or fullness of the ROI, which is jointly determined by the parent artery and the aneurysm. Our results suggested that the more regular the shape of the IAs, the lower the possibility of ISS. It should be noted that radiomics shape features may exceed traditional manually measured morphological parameters in exploring the shape regularity of IAs and parent arteries. The Elongation AUC of ROC was 0.73 and the optimal cut-off was 0.595 (Figure 3). The sensitivity of this indicator is low and the specificity is high, which helps to exclude true negatives. The ISS degree median of the group with Elongation <0.595 was larger than the ISS degree median of the group with Elongation >0.595. We can see in the box picture (Figure 4), that patients with Elongation <0.595 have a greater probability of developing more serious ISS after PED placement. The results have clinical significance in helping clinicians to preoperatively judge the risk of ISS after PED treatment for UIAs and make treatment decisions.

## Limitations

This study focused on the morphological factors predicting postoperative ISS after PED implantation for UIAs, but the sample size was small and the study was conducted at a single center. This limitation, together with the retrospective property and relatively short follow-up period, limits the extension and application of the conclusions, which need to be validated in a large multi-center prospective dataset.

## Conclusion

Elongation, defined as the regularity of the aneurysm and parent artery, is a potential risk factor associated with ISS after PED implantation for UIAs. Our results suggest that the more regular the shape of the aneurysm and parent artery, the lower the possibility of ISS.

## Data availability statement

The original contributions presented in the study are included in the article/supplementary material, further inquiries can be directed to the corresponding authors.

## Ethics statement

Ethical review and approval was not required for the study on human participants in accordance with the local legislation and institutional requirements. Written informed consent from the patients/participants or patients/participants’ legal guardian/next of kin was not required to participate in this study in accordance with the national legislation and the institutional requirements.

## Author contributions

XM and YL made substantial contributions to the conception and design of the study, and provided final approval of the version to be published. CL, JW, and YJ had the main responsibility for the experiment, the acquisition of data, and data analysis. HJ and JL wrote the article. All authors contributed to the article and approved the submitted version.

## Funding

This study was supported by the Beijing Gold-Bridge Project (Grant Number ZZ21060) and the National Natural Science Foundation of China (Grant Number 82171289).

## Conflict of interest

The authors declare that the research was conducted in the absence of any commercial or financial relationships that could be construed as a potential conflict of interest.

## Publisher’s note

All claims expressed in this article are solely those of the authors and do not necessarily represent those of their affiliated organizations, or those of the publisher, the editors and the reviewers. Any product that may be evaluated in this article, or claim that may be made by its manufacturer, is not guaranteed or endorsed by the publisher.

## Footnotes

^1^ https://PyRadiomics.readthedocs.io/en/latest/
